# Infant-adult synchrony in spontaneous and nonspontaneous interactions

**DOI:** 10.1371/journal.pone.0244138

**Published:** 2020-12-18

**Authors:** Zamara Cuadros, Esteban Hurtado, Carlos Cornejo

**Affiliations:** Laboratorio de Lenguaje Interacción y Fenomenología, Escuela de Psicología, Pontificia Universidad Católica de Chile, Santiago, Chile; University of Iowa, UNITED STATES

## Abstract

Infant-adult synchrony has been reported through observational and experimental studies. Nevertheless, synchrony is addressed differently in both cases. While observational studies measure synchrony in spontaneous infant-adult interactions, experimental studies manipulate it, inducing nonspontaneous synchronous and asynchronous interactions. A still unsolved question is to what extent differ spontaneous synchrony from the nonspontaneous one, experimentally elicited. To address this question, we conducted a study to compare synchrony in both interactional contexts. Forty-three 14-month-old infants were randomly assigned to one of two independent groups: (1) the spontaneous interaction context, consisting of a storytime session; and (2) the nonspontaneous interaction context, where an assistant bounced the infant in synchrony with a stranger. We employed an optical motion capture system to accurately track the time and form of synchrony in both contexts. Our findings indicate that synchrony arising in spontaneous exchanges has different traits than synchrony produced in a nonspontaneous interplay. The evidence presented here offers new insights for rethinking the study of infant-adult synchrony and its consequences on child development.

## Introduction

Interpersonal synchrony is defined as the spontaneous coordination of the interactants' body patterns in time and form [1–4]. When two or more individuals interact in social settings, they tend to coordinate at behavioral [5–11], physiological [12–20], and linguistic levels [21–26]. This phenomenon has been observed between adults chatting in an affiliative and argumentative way [27–29], solving joint tasks in a competitive or cooperative setting [30, 31], playing sports games [32–34], dancing [35, 36], or performing music together [37, 38]. Coordination has also been reported between pairs of preschoolers [39] and child-adult couples [40, 41] during joint drumming. Moreover, it has been described between parents and their infants in turn-taking conversation contexts [42], free play [43], and daily life routines [44, 45].

In the field of child development, the infant's ability to synchronize with their caregivers has been reported as early as the first day of life. It has been observed that newborns coordinate the movements of their limbs with adult speech [46]. Young infants can also synchronize with adults in their facial expressions [47–49], gaze direction [50, 51], vocalizations [52], body orientations [47, 53], and body movements [54, 55]. These synchronization experiences have been hypothesized to be crucial for the infants' entry into the social world [47, 49, 56]. Through synch episodes, infants start to co-regulate their endogenous rhythms [57] and to adapt to interpersonal rules [58]. Synchrony also provide the foundation for the child's later capacity for cooperation [59], symbol use [60], intersubjectivity [47], speech, and language [4, 46, 61]. Thereby, the infants' ability to achieve synchrony with adults would serve co-and self-regulatory, communicative, social-emotional, and cognitive functions [56, 57, 60, 62, 63]. This statement is supported by evidence from observational as well as experimental studies on infant-adult synchrony. Nevertheless, it is relevant to note that synchrony is addressed differently in both research lines. Here we investigate whether the differences in empirical approaches point to the same type of synchrony.

### Observed synchrony approach

Observational research has approached synchrony in terms of observable body patterns of parent-infant matching in mutual attention periods [56, 64]. In these works, synchrony is quantified from detailed coding and scoring techniques of spontaneous dyadic events, such as microanalysis or behavioral coding [64–66]. By estimating frequencies, durations, and concurrences, observational studies evidenced that baby-mother matching exhibits coherent body patterns in timing and rhythm even as early as the first day of life [46, 54, 67, 68]. In addition to computing descriptive and probabilistic measures, contemporary studies have also calculated the degree of coherence and lead-lag structure underlying the sequences of infant-parent contingencies through time series analysis [65]. Some temporal and rhythmic characteristics of synchrony have been observed to change over the first year of life. For example, the duration, lags- and direction of influence between interactants and their functions vary over time, but not the strength of the associations between the partners’ behavior—the low but significant correlations between infant-parents behaviors range from 0.16 to 0.20 [15, 58, 69].

Mother-infant coordination has been reported to increase in length and mutuality along development. Early synchronous interactions have been characterized as shorter and less reciprocal than those observed at around 9 months [56, 62]. In the earliest interactions, mothers assume an active role in adjusting and modulating infants' biological rhythms [57, 70]. They follow the infants' responses, scaffolding their physiological regulation [4, 71]. When infants become more active interaction partners at around 3- to 9-months, the lead-follow structure grows into mutual during authentic turn-taking exchanges (i.e., infant-leads/mother-follows and mother-leads/infant-follows) [15, 57, 71].

Given that synchrony relies on each partner's contribution, its presence is not constant throughout the interpersonal interaction [49]. Recurrent but intermittent shifts from mismatched states to matching states have been reported in interactions between mothers and their sons with ages ranging from 3- to 9- month-olds [72] and 14- to 18- month-olds [54]. These matching episodes occurred during less than 40% of the entire interaction time [42, 49, 56, 73]. Furthermore, less synchronized and asynchronous exchanges have been found when one or both individuals disengage from the interaction due to changes in interactional goals [74] and the physiological and psychological functioning [13, 70, 75]. For instance, dyadic synchrony is affected by infants' physiological immaturity at birth and infant negative reactivity [76, 77]. It also is impaired by physio -and psychological dysregulation of mothers related to high cortisol levels [78], low oxytocin levels [15, 79], and mood disorders (e.g., depression and anxiety) [80–83].

Observational studies suggest that synchrony in early infancy may vary according to the qualities of relationships, given the link between emotional engagement and synchrony. There were found differences in synchrony between mother-infant and father-infant [69, 71, 84]; in the same-sex infant-parent dyads in contrast to the opposite sex dyads [69, 84]; and between mothers and their twins compared to mothers and their singletons [85]. Although spontaneous interactions between infants and unfamiliar adults have been less explored, one study found that synchrony between mothers and 14- to 18-month-olds unknown infants was weaker than that between mothers and their sons [54].

As to the lead-lag structure of reciprocal relationships, it has been reported that the time lag of synch responsivity between who is leading and who follows the interaction decreases as infants develop—ranging from 3 s to 1 s, indeed reaching zero-lag [58, 69, 82, 83]. These experiences of mutual synch provide a window to shape the infant's emotional and social skills [71]. Mother-infant synchrony observed at 3 and 9 months old has been associated with secure attachment at 1 year [67, 86, 87]; self-control [73], symbolic play, and internal state talk [60] at 2 years; and empathy at 13 years old [58].

### Elicited synchrony approach

The consequences of coordinated motion for child development has also been addressed by eliciting infant-adult synchrony. These works differ from observational studies in conceptualization and measurement of synchrony, dyad composition, and target age. Most of the observational studies focus on interactions between under 1-year-old infants and their mothers or fathers. Experimental works have explored the effects of infant-unknown adult synchrony beyond the first birthday when they are growing into more active social agents [88, 89]. By 12 months old, infants understand that people's actions can be goal-directed and provided them information to reach them their aims [63, 90, 91]. At 14 months old, infants also begin to cooperate with strangers [92–94]

Experimental research has approached synchrony in terms of infant-unknown adult matching in shared movement experiences [95–97]. In these studies, an assistant sways the infant in synchrony or asynchrony according to the interaction partner's movements. The accuracy of synchrony manipulation is almost always verified by interjudge reliability—except for one work that measured and compared the acceleration of the assistant and experimenter across the conditions by using a Nintendo Wii remotes at their waists [89]. This procedure provides infants with a particular shared movement experience in form and time. The assistant and experimenter mirror their movements, thereby synchrony acquires the same morphology across time. Given that the equal cycles of a specific action are repeated at the same time intervals, steady synch in time and rhythm occurs while the activity length [89, 97].

The coordination elicited through this procedure guarantees an experience alike to that experienced in situations of musical engagement. Similar to elicited synchrony, "musical engagement involves temporal alignment of movements to evenly spaced, predictable beats" [89]. Since the prosocial effects of musical engagement in adults are well known [98–101], elicited synchrony has been used to explore the prosocial consequences of move in synch with infants who are still not good at moving to a regular beat [89]. For example, it has been found that to move steadily together with a partner guides infants' social choices at 12 months, but not at 9 months [97]. Interestingly, after being bounced to music in synchrony, 14-month-old infants enhanced helpfulness towards an unknown adult compared to those who were asynchronously bounced to the music [89, 102, 103]. The bouncing in sync improved infants' helping behavior more than asynchronous bouncing, even in the absence of music [104]. Similar results have been reported for preschoolers. Four-year-old children [59, 96] and 8–9-year-old children [105] increased their cooperation towards an unfamiliar peer following synchronous movement, compared with conditions of asynchronous movement or movement absence.

Experimental studies with adults have also reported that the underlying emotional background to social relationships precedes and proceeds to episodes of body synchronization. For example, studies with adults show that prior affective bond between two strangers impacts their subsequent bodily coordination. Thus, adults establishing positive affective ties synchronize with each other more than those who create a negative emotional bond [27, 106]. There is also evidence that following a coordinated interaction between adults, their positive feelings towards the interaction partner increase [30, 35, 107–109]. It has also been reported that synchronous interaction between children improves perceived similarity and closeness towards each other [105].

### The present study

Although both lines of research have successfully studied the consequences of dyadic synchronization, the type of synchrony addressed seems to be different. Observational studies measure synchrony as it spontaneously emerges during infant-parent interactions. In particular, they measure the temporal dimension of synchrony regardless of their form. On the other hand, instead of measuring synchrony, experimental studies manipulate it, inducing nonspontaneous synchronous and asynchronous interactions between infants and unfamiliar adults. Research on synchrony between adults shows a similar trend. Observational studies measure the temporal synchronization naturally emerging between pairs of conversing adults [27, 28, 108, 110, 111]. Recently, spontaneous morphological synchronization has also been described in adults. Coordination between conversing adults adopted two shapes: mirror-like and anatomical [112]. While mirror-like coordination implicates movements that reflect those of the partner-i.e., ipsilateral ones, anatomical coordination involves movements that reconstruct the partners’ body-scheme-i.e., contralateral ones [113, 114]. Conversely, experimental studies elicit synchrony in adults through joint tasks that require intentional or unintentional synchronization in time and form with a referent (e.g., a metronome, a pre-recorded video, or another adult participant). Examples of such joint tasks include finger tapping [107, 115], rocking in a rocking chair [116, 117], swinging pendulums [118–120], climbing stairs [106], walking [121–124] or jumping [125].

To date, no known studies have measured and differentiated the temporal and morphological dimensions of synchrony in spontaneous and nonspontaneous interactions. A key question is to what extent synchrony emerging in spontaneous interactions differs from that produced in a nonspontaneous interaction. Are the temporal and morphological dynamics of synchronous movement generated spontaneously comparable to those of being externally bounced in synchrony? To address this issue, we conducted a study to compare synchrony simultaneously in spontaneous and nonspontaneous infant-adult interactions. We measured synchrony between infants and an unfamiliar adult. We employed an optical motion capture system (henceforth: mocap) to accurately track participants' interplays. Through this device, we ensured to record, measure, and compare the participants' subtle movements rather than movements perceptible to the eye. In particular, the slight backs movements of participants were tracked while they involved in: (1) a nonspontaneous interaction context, in which an assistant bounced the infant in synchrony with a stranger; and (2) a spontaneous interaction context consisting of a storytime session, in which participants freely interact. We also asked the caregiver to report the perceived affective-state in the infant before each interactive session.

In sum, we reproduced the interaction contexts previously introduced by experimental and observational research lines but using a different method of capture and analysis in order to register the temporal and morphological differences in synchrony. Clarifying this issue is essential to a better understanding of dyadic coordination phenomena. If synchrony is different in each case, it would be suitable to introduce a denomination that corresponds to their features. Such a distinction would shed light on the complex relationship between synchronization and its consequences for social development. It would open a promising scenario to explore and discuss the specific effects of the distinct temporal and morphological characteristics of synchrony.

## Materials and methods

### Participants

Forty-three 14-month-old typically developing infants (22 girls; *M* age = 14.3 months; *SD* = 0.2 months) were recruited from Family Health Centers in Santiago de Chile city and day nurseries at the Chilean National Board of Kindergartens and the Pontificia Universidad Católica de Chile. We select 14-month-old infants due to literature indicating that at that age, they have gained some motor independence and help strangers to reach their goals [63, 89, 90, 92–94, 102–104]. All infants included in the study were able to walk independently and had parents without any diagnosis of mental disorders. Seven additional infants who participated in the study were excluded from the final analyses due to equipment failure (*n* = 1), excessive fussiness (*n* = 3), and disengagement from the interaction to look for the mother or to turn away from the storyteller (*n* = 3). The study was approved by the Ethical Committee of Social Sciences at the Pontificia Universidad Católica de Chile and the Ethical Committee of Metropolitan Health Service South-East. Informed consent was obtained from all parents.

### Design

Infants were randomly assigned to one of two independent groups: the spontaneous interaction context (*n* = 22, 12 girls) and the nonspontaneous interaction context (*n* = 21, 10 girls). While the spontaneous interaction context comprised a storytime session, the nonspontaneous interaction context included an activity in which an assistant bounced the infant in synchrony with a stranger in the absence of an audible musical stimulus [104]. We did not include audible musical stimuli in each condition to control the known power of music to induce emotions, feelings, affiliation, and musical engagement [99, 101, 126–128]. These variables have been reported to influence synchrony between interactants [30, 106, 108, 129]. In both conditions, synchrony was measured by means of mocap. All infants participated in a familiarization phase before synchrony was measured. In each condition infants took part in an interaction with one of two unknown female adults (*M* age = 21.5 years old; *SD* = 0.7 years old). Each storyteller interacted roughly with the same number of participants balanced by sex and condition (storyteller 1 = 22; storyteller 2 = 21). Both unknown adults were blind to the objectives of this study and were not acquainted with any participant. Based on reports of on the bidirectional relationships between synchrony and affect, we required all parents to report the perceived intensity of the infant’s affect-state before each interaction session by means of the Spanish version of the PANAS-C-SF [130].

### Apparatus

The body movements of interacting pairs were recorded with a mocap consisting of 36 NaturalPoint Optitrack Prime 41 cameras and a personal computer running Motive software. The cameras were positioned close to the ceiling, surrounding a rectangular perimeter (width 3 m x depth 4 m x height 2 m) in a room. The mocap tracks the position of small infrared-reflective spherical markers. We followed the recording protocol by Cornejo, Hurtado [112] to improve participants’ comfort. Seven reflective markers were positioned on each person's body: the upper back (2x), the elbows (2x), and the head (3x). We used elastic bands to sustain the small reflective markers around the adult body. In infants, we used a comfortable sweater and a baby hat bearing the markers adhered to the key positions. Position signals were recorded at 120 Hz (sampling periods per second).

### Procedure

Upon each family’s arrival at the laboratory, a female assistant executed the familiarization phase. In this phase, she followed the next sequence of actions. First, she familiarized the participants with the video and optical cameras in the room. She explained and showed the mocap functioning to parents before described the study procedure. Second, the assistant instructed in detail to parents about their role and location during the sessions. She explained to parents that they could play freely with their children during the familiarization phase, but they should avoid interrupting the interaction activity with the unknown adult. She requested the parents to remain silent and sitting behind the infants -out of their visual field-, once the familiarization phase was ended. Third, the assistant invited parents (1) to sit behind infants, who were standing up around a table, and (2) to animate them to play with toys settled on the table. The unknown adult was located on one side of the table and already had the reflective markers adhered on her body. The unknown adult was instructed to remain with a gentle attitude but without interacting directly with them. Fourth, the assistant asked the parents to dress the infant in the sweater and hat that had the markers adhered to the key positions, while he/she played. Fifth, the assistant asked parents to rate the emotional intensity perceived in the infant during their stay in the laboratory by answering the Spanish version of the PANAS-C-SF [130]. Finally, the assistant removed the toys from the table and told the parents that the interaction phase was about to begin. She reminded them to remain silent without disrupting the activity and to stay sitting on a chair behind the infants out of their line of sight.

**Fig 1:**
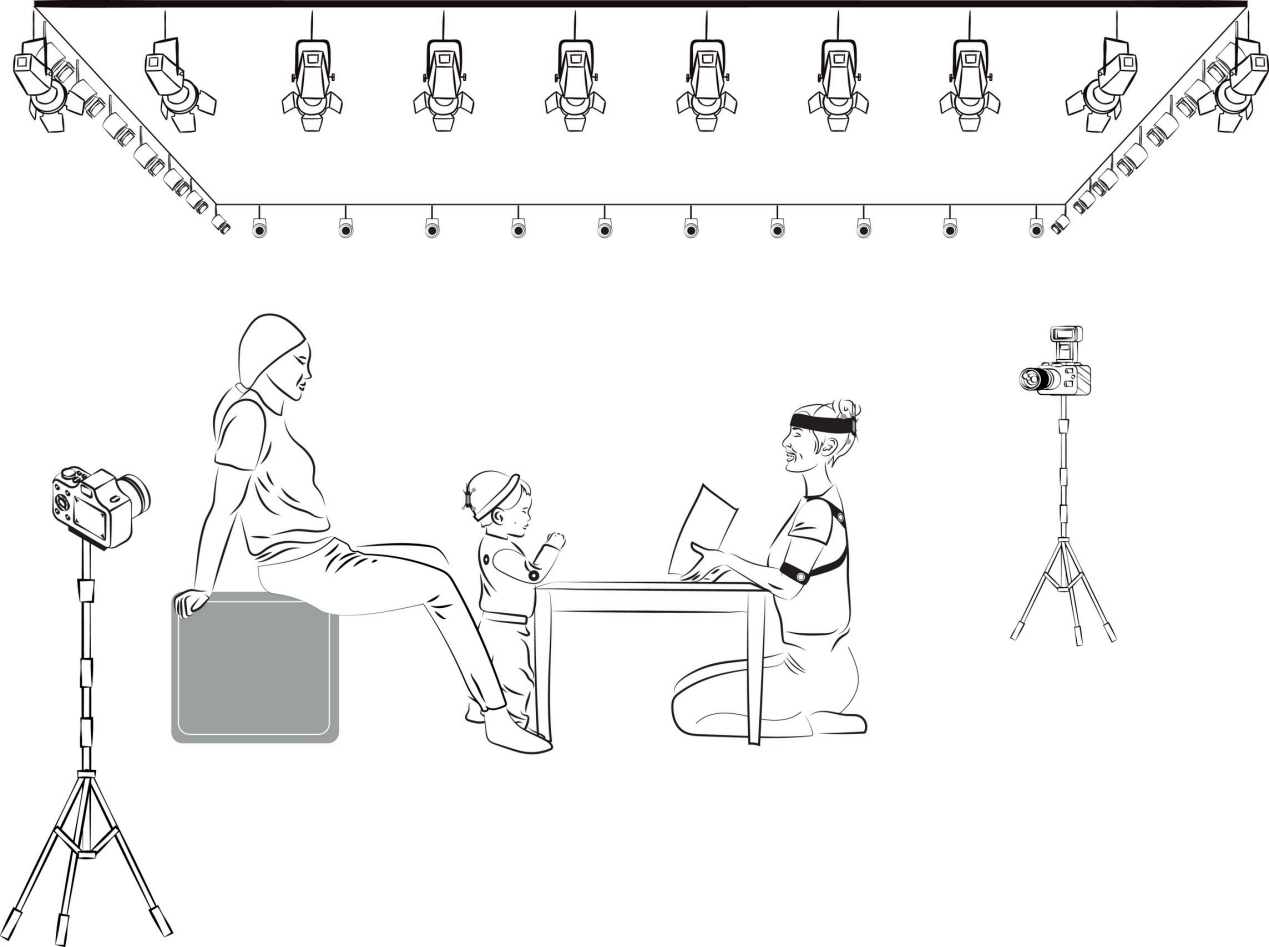
Illustration of the room setting and spatial disposition of the participants in spontaneous interaction context. 36 Natural Point Prime-41 purpose-specific cameras were positioned close to the ceiling, creating a rectangular perimeter above and surrounding the participants. During the reading session, the infant and the unknown adult were located in front of each other around a square table separating them.

After the familiarization phase, the infants took part in an interaction phase with one of the two unknown female adults. In the spontaneous interaction context, the infant engaged in a face-to-face storytime session with an unknown adult; both were located in front of each other, around a table separating them (see Fig 1). The adult spontaneously showed and told the Bérengère Delaporte’s short story “Where is Mom Elephant?” to the infant. The story narrates the search a baby elephant makes of his mother in the forest until he finds her. The session was recorded by mocap and video cameras and lasted on average, approximately 145 s (the duration range was from 126 s to 151 s).

**Fig 2:**
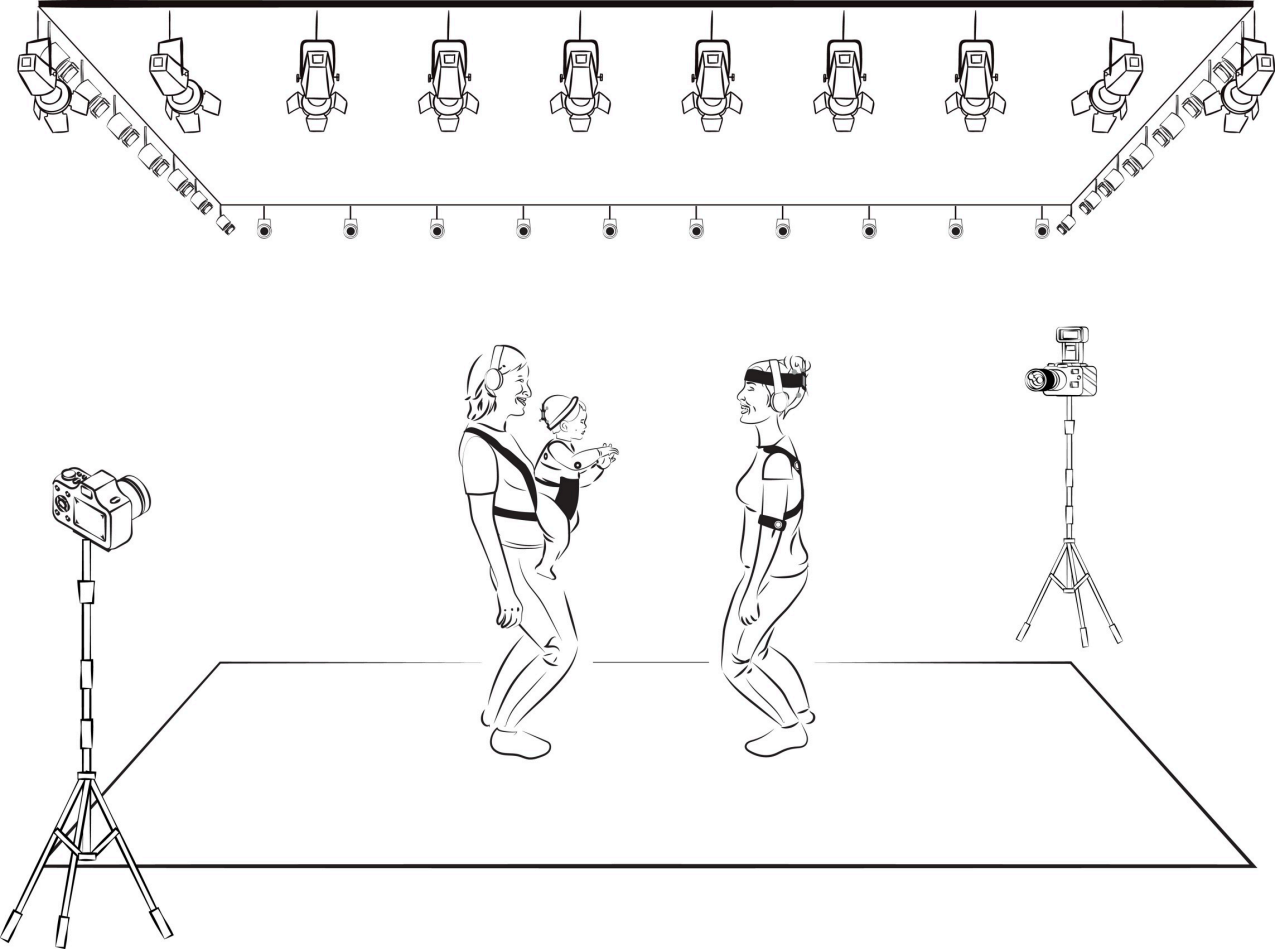
Depiction of the room setting and spatial disposition of the participants in nonspontaneous interaction context. During the bouncing session, the assistant that held the infant and the unknown adult were standing in front of each other.

In the nonspontaneous interaction context, the infant participated in a face-to-face steady swaying session with an unknown adult. We emulated the bounce in sync without music by Cirelli, Wan [104]. Thereby, the assistant held and bounced the infant in a carrier facing the unknown adult (see Fig 2). The assistant and the unknown adult bounced gently in synchrony according to a rhythmic beep to which only they were listening via headphones. The movement session lasted 145 s and was recorded by mocap and video camera.

### Preprocessing

We preprocessed the initial 120 s of each interaction following the recommended protocol by Cornejo, Hurtado [112]. Data preprocessing was performed manually. We used custom scripts to trajectorize the markers. Mocap data for each couple were labeled with corresponding body parts and identified the participant to which each marker belonged. Finally, we visually inspected the results. This decision of selecting 120 s of each interaction to analyze is consistent with the interaction times analyzed in synchronization manipulation studies, ranging from 40 to 144 s. After preprocessing, our data consisted of one time series for the positions of each interactant.

### Analysis

To quantify bodily coordination between a child and an adult, we measured linear relationships between the motion (speeds) of both by means of Pearson correlations. Those were computed both for immediacy and for several positive and negative delays. In other words, the result was a cross-correlation curve, that displays a Pearson correlation level for each of the studied time lag values, whether the infant and the adult coordinate immediately (lag = 0), or any of the two imitates motion of the other within a short time difference (lag ≠ 0). We wanted to look for significant correlation patterns with a high statistical power, so to capture even subtle coordinations. This was relevant since the possibility of much smaller coordinations in one condition than the other was embedded in our research question: we needed to find and measure spontaneous coordinations, which could be smaller and less consistent than clearer nonspontaneous coordinations from bouncing.

In order to make an efficient use of data, we followed the statistical inference strategy in Cornejo, Hurtado [112], which proceeds as follows. First, a cross-correlation curve is computed for each experimental session. This yields one Pearson correlation per session, per delay time. Second, for each delay time, correlations for all sessions are pooled into a single Pearson correlation value that corresponds to that delay (see Cornejo, Hurtado [112], section “Aggregation of Cross-Correlation Curves”, for details). By correlation pooling we mean that, in the same fashion as a regular Pearson correlation, we sum up deviation products and square deviations, but those deviations are not computed as a difference from grand averages of the two variables being correlated. Instead, we allow each session to have its own pair of average levels, so deviations are taken in the context of their own session. We use this strategy because it allows us a clean handling of the distribution of Pearson correlations, while it provides it with a single standard error to test and expand, with high statistical power, and adequate handling of the high number of degrees of freedom resulting for analyzing data at a frame to frame level. Doing this instead of averaging has the advantage of producing a number that behaves as, and effectively is, a Pearson correlation value. Therefore, it can be statistically tested as such with usual techniques. Additionally, such result is backed by a number of degrees of freedom which is much bigger than the amount of sessions (as would be the case if an average was computed), because a pooled correlation obtained this way is conceptually equivalent to a single correlation from the concatenation of all recordings in the same experimental condition. Third, this aggregate correlation in a scale from -1 to 1 is transformed into a normally distributed variable by means of a Fisher transform, which consists in applying an inverted hyperbolic tangent function to the correlation value *r*.

$$F[r]={tanh}^{-1}[r]$$ (1)

This enables the following fourth step: to test whether the correlation is significantly different from zero by a simple z test (classic normal distribution) with a standard error very close to

$$\sigma \approx \frac{1}{\sqrt{\sum_{i=1}^{m}n}}$$ (2)

where *m* is the number of recorded sessions and *n_i_* is the length of the *i*-th session in number of sampling periods [i.e. equivalent to frames in a video]. Note how standard error rapidly decreases as the number of *recorded frames* (of which there are several per second) increase, a fact that leads to a very efficient use of data. This approximation for the standard error, and the whole Fisher transform approach works well for high numbers of degrees of freedom, in contrast to usual t-tests for correlations which tend to increase type I error rate as degrees of freedom (i.e., sample size) increase.

As a result, even a small hypothetical study, with a sample size of 10 sessions, at 100 seconds each, with 10 samples per second, would yield a standard error of $\sigma \approx 1/\sqrt{10000}=0.01$. As previously mentioned, our correlations result from pooling Pearson correlations; meaning that deviations are not computed from grand averages, but from averages in each session. This allows us to extract a single standard error figure in the same fashion as the Student *t* statistic pools errors from both samples, or ANOVA error mean squares pool errors from groups or subjects with different means. Since Fisher transform is practically an identity function (i.e., does not need to be applied) for correlations of small magnitude, this means that correlations of magnitude as small as |*r*|≈0.02 (two standard deviations) can be detected with statistical significance. For this study, with around 20 sessions in each condition, at 120 second each, with a sampling rate of 120 Hz, correlations from $2\sigma \approx 2/\sqrt{288000}=0.004$ (two standard deviations of 0.002) can be regarded as statistically significant. It is important to note that, just like traditional t testing on correlations, and unlike other common inference tests, when the statistic under study is correlation, only the sample size and correlation value determine the statistical significance. Standard error is a function of those, and therefore does not need to be provided additionally. This is related to the fact that, even such small correlations would not survive the Pearson formula for such big numbers of samples if they were noise. In consequence, with such sample sizes, even these small correlations can result from a remarkable consistency.

## Results

**Fig 3:**
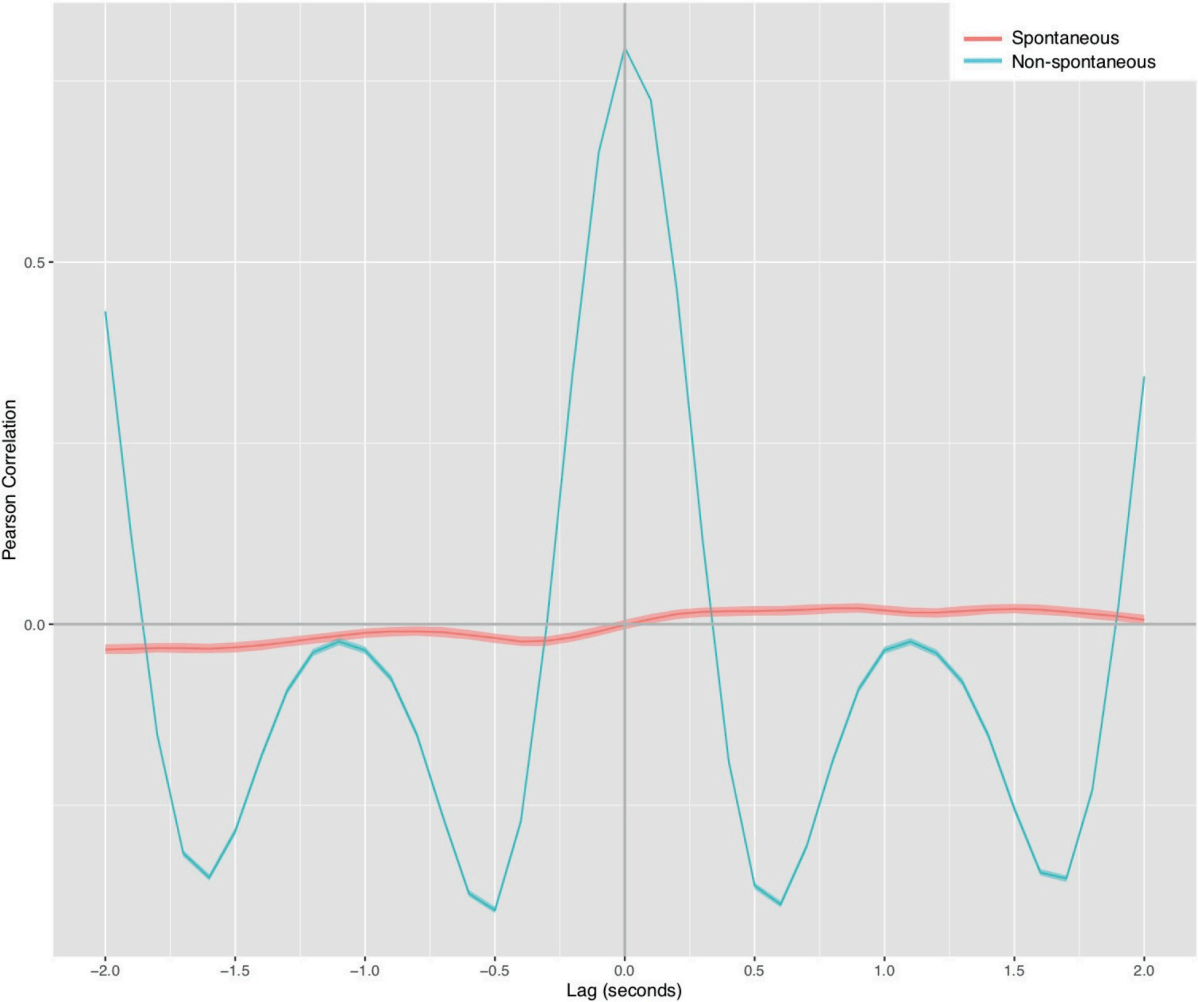
Cross-correlation curves for spontaneous and nonspontaneous interactions. The colored area surrounding the curve indicates the confidence interval. Negative lag times in the plot correspond to adult lagging behind the infant, i.e., the adult's reactions to the infant. Conversely, positive lag times correspond to the infant’s reactions to the unknown adult. Data have been organized so that positive correlation values indicate to mirror-like coordination between the interactants. Conversely, negative correlation values correspond to anatomical coordination between interactants.

Fig 3 shows cross-correlation curves for the motion data captured in spontaneous and nonspontaneous interactions. We tested differences with respect to zero Pearson correlation values in cross-correlation curves. Around each cross-correlation curve was plotted a confidence interval (see the colored area surrounding the curves). The correlations are statistically significant where their confidence intervals do not touch the zero-correlation horizontal line. We used the Fisher transformation for p-values and the confidence interval computation based on a Holm-Bonferroni correction for the 41 correlation values in a cross-correlation curve to control for the family-wise error rate. The alpha level was set at 0.001.

When comparing both conditions in Fig 3, we observed correlations with different magnitudes and morphologies. As the confidence intervals displaying around the curves do not overlap or touch the zero-correlation horizontal line, the correlations reveal statistically significant differences between the spontaneous and nonspontaneous conditions. The exceptions are a delayed coordination performed by the adult (*t* = -0.3 s, *r* = -0.000, *p* = 0.784) in the nonspontaneous condition and zero-lag coordination in the spontaneous condition curve (*t* = 0.0 s, *r* = -0.001, *p* = 0.685). Conversely, zero-lag coordination in the nonspontaneous condition is the highest correlation peak observed in Fig 3. This peak corresponds to simultaneous coordination between an adult and the infant being externally bounced in synchrony (*t* = 0 s, r = 0.80, *p* < 0.001), i.e., they move in a similar fashion and at the same time. The oscillating shape adopted by this curve is a consequence of the high consistency of the simultaneous coordination. Because of the strong general periodicity and similarity of both motion signals, the time shifting used to compute cross-correlations produces an alternation between lags at which signals are similar and lags at which they are opposite, with zero-lag corresponding to highest similarity.

**Fig 4:**
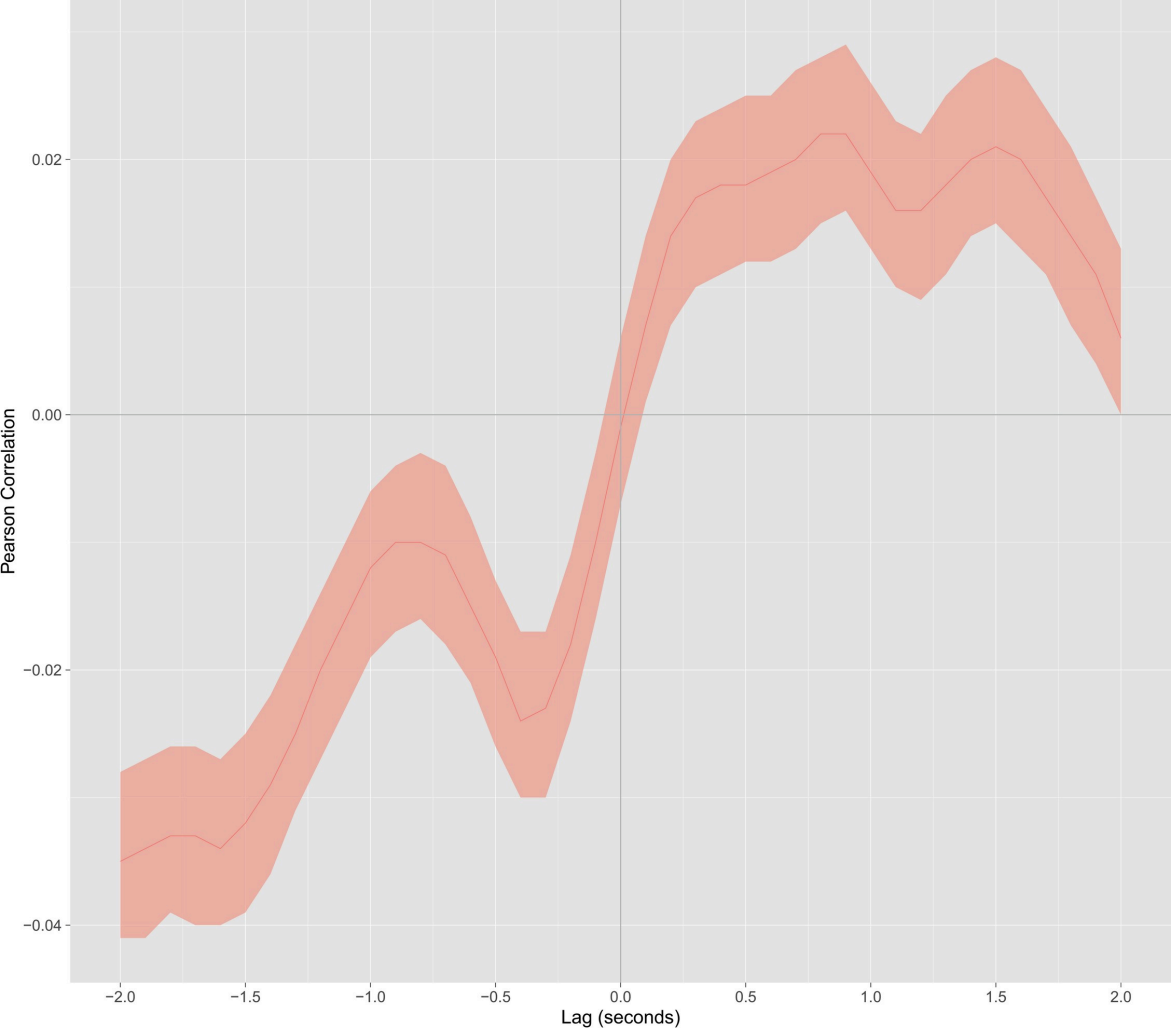
Cross-correlation curve for spontaneous interaction. The colored area surrounding the curve indicates the confidence interval. Negative lag times correspond to adult lagging behind the infant. Positive lag times correspond to the infant’s reactions to the unknown adult. Positive correlation values indicate to mirror-like coordination between the interactants. Negative correlation values correspond to anatomical coordination between interactants.

Fig 4 zooms in on the cross-correlation curve for the spontaneous condition. In the positive quadrant of the upper half-plane, the curve shows a mirror-like coordination pattern between the participants. This result means that positive correlation values represent coordination where the infant tends to display a motion pattern reflecting the adult motion but later (e.g., the interactants' backs approach each other). Two mirror-like correlation peaks are evident in the curve: first at *t* = 0.9 s (*r* = 0.022, *p* < 0.001) and second at *t* = 1.5 s (*r* = 0.021, *p* < 0.001). These peaks correspond to the infant’s reactions to the adult with a 0.9 s and a 1.5 s lag, respectively. In the negative quadrant of the lower half-plane, the curve displays an anatomical coordination pattern between interactants. Negative correlation values denote that adult's movements reproduce the infant body-scheme with a lag (e.g., reverse motion between participants' backs). Two anatomical correlation peaks are evident in the curve: first at *t* = −0.4 s (*r* = -0.024, *p* < 0.001) and second at *t* = −1.6 s (*r* = -0.034, *p* < 0.001). Both peaks indicate the adult tends to imitate the infant with a 0.4 s and a 1.6 s lag, respectively.

Table 1 displays a comparison between cross-correlation values for the spontaneous and nonspontaneous conditions based on the times at which their main peaks occurred. A linear mixed effects model was fitted on the values of the simultaneous and delayed coordination (z scores) in both conditions, controlling for infants' sex and the unknown adult who interacted with them. The main effect of the condition still reached significance on correlation values (*F*_(1, 39)_ = 382.39, *p* < 0.001). However, no main effects of covariates on the values of simultaneous and delayed coordination were found. There was no statistically significant effect of infants' sex (*F*_(1, 39)_ = 0.55, *p* = .74) and the adult who interacted with them (*F*_(1, 39)_ = 1.76, *p* = .15) on correlation values. Finally, an analysis of variance (ANOVA) on caregivers’ reports reveals that the scores of infant's affect did not differ significantly across conditions, both positive (*F*_(1, 41)_ = 3.43, *p* = .071) and negative (*F*_(1, 41)_ = 0.20, *p* = .66) affect.

**Table 1:** Peaks of cross-correlation values for spontaneous and nonspontaneous conditions.

	Spontaneous interaction				Nonspontaneous interaction			
Time-lags	*r*	*SE*	*z*	*p*	*r*	*SE*	*z*	*p*
-1.6 s	-0.03	0.00	-0.03	<0.001	-0.35	0.00	-0.37	<0.001
-.4 s	-0.02	0.00	-0.02	<0.001	-0.27	0.00	-0.28	<0.001
Zero-lag	0.00	0.00	0.00	0.685	0.80	0.00	1.09	<0.001
.9 s	0.02	0.00	0.02	<0.001	-0.09	0.00	-0.09	<0.001
1.5 s	0.02	0.00	0.02	<0.001	-0.26	0.00	-0.26	<0.001

## Discussion

### Time and morphology of synchrony

The current study shows differences between synchrony accurately measured in spontaneous and nonspontaneous infant-adult interactions. Our findings indicate that synchrony emerging in spontaneous interactions differs in time and morphology from that elicited in a nonspontaneous interaction. This finding explains the ample differences in the magnitude of cross-correlation. In comparison to the induced synchrony, spontaneous coordination phenomena look very marginal; yet they are statistically significant. With the exception of synchronized bouncing in the nonspontaneous condition, our correlation values are indeed of small magnitude. This responds to the fact that coordinated motion between two people is often just a small part of total motion. Therefore, the high statistical power of our method is relevant to sensitize and test subtle coordination usually buried under bigger uncoordinated motion signals. Correlation works because it detects consistent linear relationships and is not affected too much by inconsistent (or some nonlinear) relationships. For instance, we found a couple of statistically significant correlations with values around |*r*|≈0.02, which corresponds to a shared variance proportion of *R*^2^ = 0.04% with respect to the whole motion of the two people under measurement. At such levels we are looking at very small coordination phenomena, hard to detect by the naked eye, both because of its little magnitude and because it can be consistently spread across considerable time within an experimental session, as opposed to easily identifiable single and clear coordination events.

Concerning time, we found higher and statistically significant zero-lag correlations in the nonspontaneous condition, compared to the spontaneous condition. This finding indicates that nonspontaneous synchrony manifests a simultaneous and temporally constant pattern of the same bounce movement throughout the 120 s interaction, hence the sinusoidal form adopted by the curve. The procedure used to synchronize participants generates this pattern. Bouncing at a constant rate produces a high correlation peak, indicating continuous and perfectly coordinated movements between partners.

By contrast, in the spontaneous interaction, synchrony at zero-lag was of a low magnitude and statistically not significant. This result accords with previous findings of low magnitudes in the zero-lag correlation of participants’ movements involved in natural interactions accurately measured. For example, a statistically nonsignificant correlation was reported in a sample of 10 adult couples debating for 10 minutes on political, social, and personal topics [27]. The lack of statistical significance was attributed to the sample size. Other studies also reported low but statistically significant correlations in samples from at least 14 couples spontaneously interacting from 10 to 15 min. In particular, zero-lag correlations were reported between adults chatting [28, 108, 112, 131]. Research using behavioral coding to study infant-parent synchrony during 5 min free-play have also evidenced low but significant correlations at zero-lag [15, 58, 69].

We assume that low synchrony at zero-lag can be related in our study to the duration of the interaction. We analyzed 120 s of spontaneous interaction to match the typical duration of the synchrony manipulation, i.e., from 40 to 140 s [89, 97, 102–104]. We did not increase the interaction time to avoid the risk of generating excessive fussiness in infants who were held and bounced in a carrier by an assistant. Most likely, the observation time recorded in the current study was not wide enough to observe the emergence of simultaneous coordination in the spontaneous condition. The differences found at zero-lag on spontaneous and nonspontaneous synchrony accurately measured seem to add evidence to the hypothesis that coordination in natural interactions is discontinuous temporally. Studies on interpersonal coordination in face-to-face social encounters show that synchronous co-activity emerge swiftly and for brief periods [3, 46, 54, 66, 132].

Concerning the form of spontaneous synchronization, we found that infants tend to mirror the adult’s motion, while adults coordinate with infants’ motions in an anatomical way. Similar forms of delayed coordination were reported in a study on the relationship between synchrony and trust in natural conversations [112]. In this study, participants mirrored the assistant’s movements at 1.1 s, and the assistant was anatomically coordinated with participants at 1.3 s. Accordingly, time delays between infants and the adult as well as the difference in the coordination morphology reported here can be attributed to two factors. First, the infants and the adult did not know each other, which manifests in longer time-delays than those reported in friends’ natural interactions [108, 112]. Second, the anatomical coordination from the storyteller towards participants may indicate the interference of her role limitating a fully natural involvement with the infant. There exists indeed evidence that synchrony patterns change importantly when one of the two participants play a confederate role, resulting in a disengagement from the interaction partner while attending to her role demands [35, 112]. This interpretation is supported by evidence from research on the development of imitation. Studies in this field show that preschoolers, adolescents, and adults tend to imitate in a mirror-like way rather than anatomically [113, 133, 134]. However, anatomical imitations can be related to cognitively demanding interactions [135, 136], whereas mirror-like imitations can be involved in affectively guided interactions [113, 137]. Presumably, the anatomical coordination of the adult towards infants suggests the prevalence of her role as a member of the research team. However, we do not have evidence that allows us to confirm this conjecture. Future research could include not only measures of the cognitive and affective involvement of team members interacting with participants but also other measures to ensure greater naturalness in interactions.

Synchrony in spontaneous interactions offers different information than synchrony in nonspontaneous interactions. In the nonspontaneous synchronization, the same motion is repeated in a simultaneous way. Thus, it is only possible to calculate the strength of the association between the temporally and morphologically constant movements of participants, i.e., the magnitude of correlations at zero-lag. In the spontaneous interaction, synchrony occurs with different time-lags at the millisecond scale. As a consequence, it can be computed the magnitude of correlations at different time-delays, including the zero-lag. The additional information on the temporal dimension of synchrony in spontaneous interactions allows also the estimation of the form adopted in simultaneous and delayed coordination as well as the lead-follow structure. Concerning the form of spontaneous coordination, we found that it could be anatomical or mirror-like. Regarding the lead-lag structure of infant-adult spontaneous coordination, we observed bidirectionality. While the infant tends to lead the interaction, the adult follows, and vice versa. The magnitudes of the delayed coordination we observe are low but statistically significant. Although these magnitudes are lower than those found in observational studies [15, 58, 69], they concur with reports of studies accurately measuring synchrony between chatting adults [108, 112, 131]. The coincidence between our findings and research with adults may be due to the nature of the capture and analysis methods used.

### Distinguishing macro- from micro-coordinations

As a whole, our results suggest that the term ‘synchrony’ covers different interactional phenomena, observable at distinct scales. On the one hand, in nonspontaneous synchrony emerge very large coordination phenomena, easily identifiable because of its high magnitude, and temporally and morphologically simultaneous and stable pattern across the movement session. This macro-coordination emerges once interactants deliberately agree to move equal in unison by a defined time [95, 138]. Numerous studies have already informed macro-coordination phenomena between adults, children, and children-adult occurring during a variety of interpersonal activities oriented towards a common goal. For instance, there are reports of macro-coordination during events that are highly structured and require practice, such as joint music performance [40, 98, 139], rule games [30, 140, 141], sports [34, 142, 143], and dancing [36, 144, 145], among others.

On the other hand, spontaneously produced coordination can hardly be detected by the naked eye, both because of its low magnitude and because it accounts for only a fraction of the total motion of people interacting. Micro-coordination phenomena emerge swiftly, for brief periods, and discontinuously in time and morphology during spontaneous and less-structured situations that do not seem to require practice and conscious effort [1, 46, 54, 146]. The features of micro-coordination have already been reported by numerous studies on interpersonal synchrony between adults, and importantly, also on adult-infants synchrony, performed in natural and semi-natural interactional contexts [15, 46, 54, 58, 60, 69, 73, 108, 112, 131, 147].

The differences in synchronous movements between spontaneous and nonspontaneous infant-adult interactions suggest strongly that both coordinative phenomena should be conceptually distinguished. While spontaneous synchrony reveals the emergence of micro-coordination, nonspontaneous synchrony elicits macro-coordination phenomena. As a result, the evidence presented here suggest that the use of the same term (‘synchrony’) is covering differing types of coordination. We consider that macro- and micro-coordinations can be suitable terms to conceptually refer to two synchronous phenomena with different temporal and morphological scales. However, other distinctions based on their distinctive features could be useful for this purpose.

The distinction between the two types of synchrony may be useful to clarify the social effects of synchrony. Both kinds of coordination have demonstrated to be effective in influencing infants' emotional and social skills [71, 95]. For example, the self-propelled infant-parent synchrony has been shown to improve the child's development of secure attachment [67, 86, 87], self-control [73], and internal state talk [60] at 2 years. Similarly, the passively experienced synchrony with an unfamiliar adult has increased infant prosocial behavior [89, 102–104]. However, we still do not know much about the prosocial effects of self-propelled synchrony, i.e., micro-coordinations. We also know too little about the emotional consequences of elicited synchrony, i.e., macro-coordinations. The distinction between micro- and macro-coordinations could be useful to investigate and discuss whether the way in which each synchrony impacts social and emotional interaction skills is the same. Thereby, an issue to solve is if each coordination is related to other variables that may precede or follow them (e.g., emotional engagement, perceived similarity, affective involvement) and how that relationship explains the social or emotional consequences. As a result, distinguish micro- from micro-coordinations could have additional implications for the methodology synchrony research, as well as for the interventions build on their results.

### Limitations and future directions

Some study limitations should be mentioned that may be pondered in future researches. First, we explored synchrony from subtle torso movements intending to analyze the temporal and morphological dynamics of the same type of matching behaviors across the conditions. Previous reports indicate that this area of the body is highly sensitive to changes in interactions of participants, which makes it an objective marker of behavioral synchrony [30, 148–154]. Due to our focus on the torso motion, other behavioral and biological markers sensitive to interactional synchrony were not considered. Synchrony can in fact be measured at different levels [146], such as psycho-physiological (e.g., galvanic skin response [13, 155], heartbeat rate [20, 70], and breath rate [4, 156]); neurophysiological (e.g., alpha, beta, and gamma frequency bands [157, 158]); linguistic (e.g., vocal rhythm and tone [52], utterance length [159], and speaking rate [160]); and behavioral (e.g., body limbs motion [2, 46, 119, 120], gaze direction [50, 51, 161], facial expressions of emotions [42, 84, 85] and gestures [2, 3, 162]). Future work should take into account these synchrony levels, which may require a combination of different data capture and analysis strategies and other interactional contexts.

Second, we used the storytime session and the bounce in sync as operationalizations of spontaneous and nonspontaneous interactions respectively. While the former was chosen for its naturalness, since it is an ordinary activity for children, the latter replicated previous studies [89, 102–104]. Nonetheless, other feasible spontaneous and nonspontaneous contexts could have been thought. In particular, different coordination patterns are likely to be found when comparing the described nonspontaneous interaction, with other spontaneous interactional situation such as a dance session to the rhythm of the music. A dance session, although natural for children and physically more similar to the nonspontaneous bounce in sync condition, implies nevertheless music as an additional variable, which we planned to control in both conditions of this study (see *Design* section). Although previous research has successfully studied the effects of elicited synchrony in the presence or absence of an audible musical stimulus for interactants [89, 97, 102–104], more research is needed to separately explore the role of music on the emergence of synchrony patterns during infant-adult spontaneous and nonspontaneous interactions. A still open question is to what extent vary the temporal and morphological dimension of synchrony in musical and nonmusical interactional contexts. For example, a particular unsolved question is whether the rhythmicity of repetitive behaviors in spontaneous and nonmusical interactions (e.g., joint walking or jumping) can be considered as having similar features to those underlying at spontaneous and nonspontaneous musical engagement.

Third, although spontaneous interaction consisted of a naturalistic book-reading session, it is still an activity framed in an experiment and conducted in an unknown place for the participants. We did not measure synchrony in the participants' houses, given the mobility restrictions of the mocap and the need for control. However, the proposed spontaneous situation offered a setting as close as possible to a natural interaction between an infant and an adult who reads the story. In addition to microanalysis, other recent technologies (e.g., Motion Energy Analysis [110], Frame Differencing Method [27], Facial Thermal Imprints [14]) can be used in future studies to capture synchrony in more natural activities and situations accurately.

Fourth, infants interacted with an unknown female in both conditions. Therefore, caution is needed before generalizing our findings to other relationships, specially the infant-parent ones. As we reviewed previously, synchrony is very sensitive to the psycho-physiological characteristics of each partner, as well as to the affective qualities and interactive modalities that are built during daily interactions with others. Findings even suggest differences in synchrony between mother-infant and father-infant interactions [69, 71, 84]. After the first birthday, infants and their parents would have had enough time to practice and adjust their physiological and behavioral rhythms [57]. Indeed, they would have had to become intimately familiar with each other's gestures and behavioral rhythms [54]. Furthermore, because of the affective reciprocity and the particularities shaping each close relationship, infants' motivation to interact with their acquaintances may vary [42] and, of course, differ from the motivation to interact with an unknown woman whom she/he had just met. For these reasons, a highly plausible hypothesis is that infant-unknown adult synchrony will be slighter than that between mothers and their sons. Despite this comparison exceeds the scope of this article, we point out the convenience of address this issue in future studies.

Finally, we found that infants’ affective states preceding the interaction with the assistant did not influence the synchrony levels during the spontaneous and nonspontaneous conditions. Nonetheless, we did not measure whether affect levels changed across the conditions. Previous studies have reported that factors related to affiliation influences synchrony and vice versa. For example, people who do not like each other tend to be less in sync [106, 163] than people who like each other [108, 121]. Research also show increases in variables related to affect and affiliation between adults following a nonspontaneous synchronization, positive affect [30], social bonding [35], and trust [109]. Synchronous interactions between preschoolers improve perceived similarity and closeness towards each other [105]. Thus, questions remain open for future research: Do infants’ affects change after a spontaneous or a nonspontaneous interaction with an unknown adult? Are there differences in affect changes between spontaneous and nonspontaneous interactions? Do changes in participants’ affects influence the synchrony levels? Future research has the challenge of finding new ways to objectively measure affect-states of overcoming the widely known problems of social desirability inherent to self-reports and the reliability of observational measures. Novel ways of accurately measuring affect could favor the study of the possible relationships between synchrony and emerging affect in interactions between infants and adults.
